#  Association of *CYP1A1 rs1048943* Polymorphism with Prostate Cancer in Iraqi Men Patients

**DOI:** 10.31557/APJCP.2019.20.12.3839

**Published:** 2019

**Authors:** Wisam H Hoidy, Ferdous A Jaber, Mohammed A Al-Askiry

**Affiliations:** 1 *Department of Chemistry, College of Education, *; 2 *Department of Biochemistry, College of Medicine, *; 3 *Department of Medical Biotechnology, College of Biotechnology, University of Al-Qadisiyah, Al-Qadisiyah City, Iraq. *

**Keywords:** Prostate cancer, CYP1A1, rs1048943, genotype

## Abstract

**Objective:**

The purpose of this study was to evaluate the relationship between *CYP1A1* gene *rs1048943* polymorphism and the risk of Iraqi men with prostate cancer.

**Methods:**

In this research, we conducted a population-based approach that intersects high-throughput genotype information from different population of Iraq to estimate the frequency of genotypes associated with prostate cancer responsivenessOur study included a total of 100 patients and 150 healthy controls. *rs1048943* genotyping has been investigated in Iraqi men in connection with prostate cancer.

**Results:**

We observed that individuals with the *rs1048943* GA genotype had an increased risk of prostate cancer relative to those with the AA genotype ( OR 95% CI of 0.449 :95%CI 0.23-0.90; P = 0.002). We found in the dominant model that the *rs1048943* GA and GG genotype displayed an increased risk of prostate cancer relative to the AA genotype ( OR 95% CI of 0.680 :95%CI 0.4-1.17; P = 0.018).

**Conclusion:**

Polymorphism RS 1048943 in the *CYP1A1* gene is associated with the risk of developing prostate cancer and is possibly one of the most significant factors in its development.

## Introduction

The prostate is a male-only gland. It is situated in front of the rectum and under the bladder of the urine. Prostate size varies depending on age. It’s about the size of a walnut in younger men, but in older men it can be much larger.. The role of the prostate is to make some of the fluid that supports and nourishes semen cells (ACS, 2012). Prostate cancer is the most common malignant urinary system tumor in Western men, the second most common cancer, and the world’s third leading cause of tumor-related deaths.Globally, nearly 1.3 million newly diagnosed cases of prostate cancer and 359,000 related deaths are expected to occur in 2018 (Bray et al., 2018; Center et al., 2012). To make the disease a major male health problem. Both environmental and genetic factors influence the occurrence and development of prostate cancer. Prostate cancer risk factors include gender, lifestyle, family inheritance, and hormonal status, but the exact etiology is not clear (Stein and Flanagan, 2010). The Indian cohort study confirmed the association of *CYP1A1* *rs1048943* with a lower risk of Prostate Cancer (Vijayalakshmi et al., 2005), this finding strongly suggests that *CYP1A1* polymorphism may be closely linked to prostate cancer susceptibility (Wei et al., 2019). Androgens are male sex hormones released by the testis and adrenal glands that play a vital role in male reproductive system and sexual behaviour. Androgens are important for prostate growth, function and maintenance (Ficarra et al., 2010). Despite the high morbidity of prostate cancer, its etiology is still poorly understood (Hsing et al., 2006). Carcinogenesis of the prostate is a complex, multi-stage and multifactor process involving several factors. Ageing, ethnic origin, and prostate cancer family history are the only risk factors that have been identified (Coughlin and Hall, 2002). The roles of environmental and genetic factors have been reported in the etiology of this disease. It is estimated that genetic factors are involved as much as 42 percent of the risk of prostate cancer (Safarinejad et al., 2012). Genetic polymorphisms of many genes, such as estrogen receptor genes, have been linked with prostate cancer (Ziad et al., 2015). The 2008 Iraqi Cancer Board recorded that prostate cancer is one of the top 10 cancers affecting men in Iraq, where new cases registered in 2008 were 246 by 3,73 and an average infected incidence of 1,56 per 100,000 population were registered either in the capital Baghdad, with 66 new cases in the same year (Khalidah, 2012). In this study, we investigated the relationship in the *CYP1A1* gene between this polymorphism (*rs1048943*) and prostate cancer risk. Our study was conducted in a case-control sample of Iraqi men based on population. We also examined whether the polymorphism is associated in the prostate with altered gene expression. 

## Materials and Methods

*Patients and Clinical Samples*

In this study, 100 blood samples were taken from patients with prostate cancer at different stages, These samples were obtained from Tumor center / Al-Diwanyah Teaching Hospital / AL-Diwanyah. One hundred and fifty blood samples were collected from apparently healthy people, The ages were similar between patients and healthy people. In a containing tube of ethylenediaminetetraactic acid (EDTA), we obtained 2 mL of peripheral blood. The Blood samples are stored at - 20 ° C until they are used in the laboratory (Ferdous and Wisam, 2018).

*DNA Concentration and Purity measurement*

The concentration and purity of extracted DNA was determined using the nanodrop, 2 mL of DNA was mounted to the nanodrop lens and measured at a wavelength of 260/280-nm; the result appeared on the nanodrop attached laptop screen. After each sample, the nanodrop lens was washed with distilled water and cotton swab; accordingly, the other samples were weighed.

*Polymerase Chain Reaction*

The technique of polymerase chain reaction (PCR) was used by Alfa Gene (Ottawa, Canada) to identify and amplify the 206-base pair (bp) band of the *CYP1A1* gene *rs1048943*. The primer was lyophilized, dissolved in a free ddH2O to give a final concentration of 100 pmol / mL (as a stock solution) and kept as a stock in -20 C to prepare a concentration of 10 pmol / mL as a working first resuspended in 90 mL of free ddH2O stock solution to reach a final volume of 100 mL (10 pmol). The total PCR reaction volume was 20 mL; the components of the reaction are outlined in Table 2. The PCR sample was then separated into a 0.5X tris / borate / EDTA buffer with 2% agarose gel at 50 V for 45 minutes by taking 5 mL from each sample. Agarose gels have been treated for 20 to 30 minutes with 0.5 mg / mL ethidium bromide. Using electrophoresis, the DNA bands are visualized and collected to record the detected bands by a gel documentation method. Reaction to amplification according to the system used shown in Table 3.

*Statistical Analysis*

Genotype and allele carrier frequencies are specified as the percentage of the total number of individuals carrying the genotype and allele. The *X*^2^ and P values < .05, the odds ratio, and their 95 percent confidence interval measures of SPSS 23 (IBM Corp, Armonk, NY were used to compare the occurrence of discrete variables between prostate cancer patients and individuals monitored.

**Figure 1:**
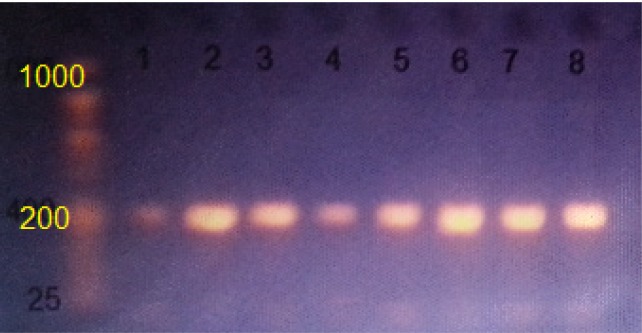
PCR Product of *CYP1A1 rs1048943* the Band Sized 206bp. The product was electrophoresis on 2% agarose gel at 50 volt, 0.5x TBE buffer for 45 min. M: DNA ladder (25-1000bp), under U.V light after staining with Ethidium Bromide

**Table 1:** Primer Designed to Amplify Polymorphism of the *CYP1A1* Gene *rs1048943*

Gene Name	SNP name	Sequence (5 ’ – ˃3’)	Tm (ºC)
*CYP1A1*	*rs1048943 *	F- 5´-CTGTCTCCCTCTGGTTACAGGAAGC-3 R- 5´-TTCCACCCGTTGCAGCAGGATAGCC-3´	62

**Figure 2:**
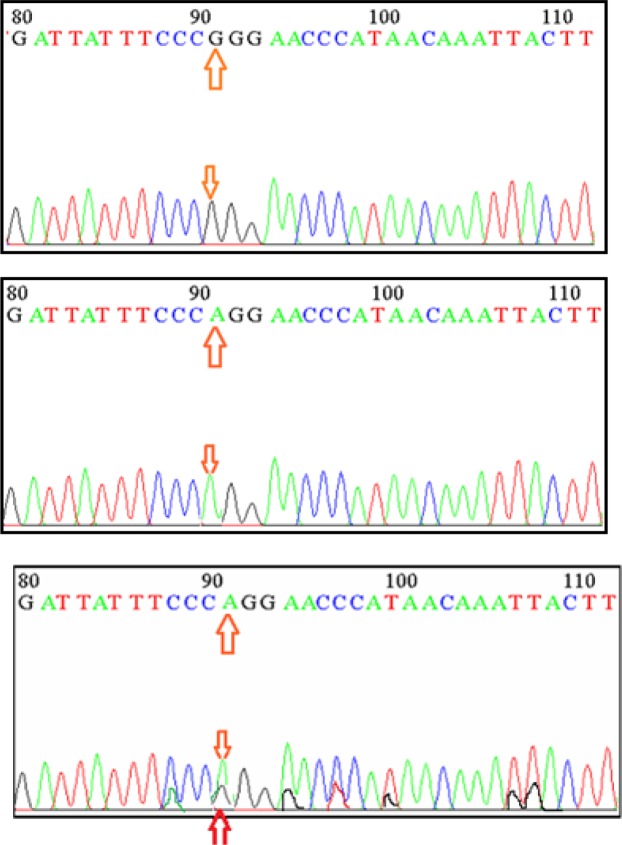
Nucleotide Sequence Chromatogram Representing Genotype of *CYP1A1* Gene

**Table 2:** Polymerase Chain Reaction 206- Base Pair Band Amplification Components

Component	Reaction size
Template DNA	5 µL
Primers (10 pmol/mL)	1 µL R 1 µL F
Deionized water	13 µL R

**Table 3:** PCR Amplification Program of Genes

Name of Gene	Cycle conditions					
	Initial denaturation	Denaturation	Annealing	Extension	Final Extension	Final hold
*CYP1A1*	95 C	95 C 30 sec	62 C 30 sec	72C 50 sec	72 C 10 min	4 C-∞*
*Rs1048943*	5 min	Repeated for 40 cycles				

∞ * means infinite

**Table 4:** The Genotypes and Allele Distribution of *CYP1A1* Polymorphism in G1 and G2

Polymorphisms CYP1A1 (G/A) Rs1048943	G1 (Control) N=150 (%)	G2 (Patients) N=100 (%)	χ2	P value	OR (95%CI)	P value
AA	92 (61.3)	70 (70.0)	6.534	0.038*	1.0 ref (1.0ref)	
GA	41 (27.3)	14 (14.0)			0.449 (0.227-0.887)	0.02*
GG	17 (11.3)	16 (16.0)			1.237 (0.584-2.619)	0.578
A allele	225 (75.0)	154 (77.0)			1.0ref (1.0ref)	
G allele	75 (25.0)	46 (23.0)	0.262	0.609	0.896 (0.589-1.64)	0.609
AA	92 (61.3)	70 (70.0)			1.0 ref (1.0ref)	
GA&GG	58 (38.6)	30 (30.0)	1.976	0.16	0.680 (0.396-1.166)	0.018*
GG	17 (11.3)	16 (16.0)			1.0 ref (1.0ref)	
GA&AA	133 (88.6)	84 (84.0)	1.14	0.286	0.671 (0.322-1.400)	0.067

## Results

Genomic DNA of adequate quality and quantity from 100 blood samples from men with prostate cancer and 150 healthy (control) men from Al-Diwanyah Teaching Hospital in southern Iraq was collected. Extension of the target sequence of *CYP1A1* *rs1048943* from these archival samples resulted in 206 bp products. The amplified fragment, which produced a single band of the desired product with a molecular weight of genes, appeared sharp in agarose gel using a technique of gel electrophoresis and packed with a DNA ladder of 25 to 1,000 bp (Figure 1). For sequence analysis, two hundred and fifty PCR product samples were sent, 20 mL of PCR product was sent for each sample, as well as 100μL (10 pmol) from the forward primer. The specimens are processed using a NICM / USA Company Applied Biosystems System AB13730XL (Figure 2). BLASTN was used in the National Center for Biotechnology Information (NCBI) to analyze the results of the sequence analysis. The findings are compared with Gene Bank data available online at the NCBI. To determine whether *CYP1A1* (A > G) *rs1048943* was associated with prostate cancer susceptibility. We analyzed these SNPs in 150 healthy individuals and 100 prostate cancer patients living in this region in the Iraqi male population living in southern Iraq. Polymorphic analysis revealed that all 3 possible genotypes ( AA, GA, GG at *rs1048943*) could be detected for these SNPs. The AA genotype was the major genotype at the *rs1048943* locus in the subjects studied. The results revealed an important correlation between *CYP1A1* *rs1048943* A, G-alleles and prostate cancer risk where *X*^2^ is 6.534 and P=.008 (Table 4). It means that this SNP and prostate cancer have a significant relationship. The results also show that the frequencies in group 1 (G1; control) were 61.3% for AA, 27.3% for AG and 11.3% for GG. The frequencies in group 2 (G2; patients) were 70% for AA, 14% for GA, and 16% for GG. There was an important correlation between G1 represents control (healthy) and G2 represents patients (P < 0.05) in *rs1048943* polymorphism of *CYP1A1* as shown in Table 4. In G1, the frequency of an allele (A) was 75% and the (G) allele was 25% and in G2 the frequencies were 77% and 23% respectively for A and G alleles. Descriptive statistical analyzes showed that the polymorphism, GA and GG genotypes of *rs1048943* were not significant compared to the genotype of AA (P = 0.160 > 0.05), There was also no significant difference between the GA and AA genotypes compared to the GG genotype (P=0.286 > 0.05). As shown in Table 4, there was also no significant relationship between the G allele and the reference A allele (P=0.609).

## Discussion

in Iraq Prostate Cancer is one of the most common cancers, its etiology has never been investigated in this country, especially the genetic risk factors. This study focused on *CYP1A1* *rs1048943*, which plays a key role in prostate cancer, and this polymorphism was associated with several types of cancer in various populations (Xueru et al., 2016). It is difficult and problematic to identify prostate cancer pathophysiology and consider its causal factors. The evidence shows that both environmental and genetic factors contribute to the development of prostate cancer (Maryam and Zahra, 2016). Smoking, diet, obesity, increased age, family background and genetic factors are the major risk factors for this disease. Prostate cancer is rare before age 45, but in older men the prevalence increases (Yang et al., 2006). Men with first-degree prostate cancer relatives have a double risk of developing cancer compared to men with no family history. Persons with two first-degree relatives with prostate cancer are at five-fold risk. According to the other research, the results of this study indicated an important relationship between the GA genotype as a mutant homozygous genotype and the risk of prostate cancer relative to individuals with the homozygous genotype AA. There is a significant correlation between the presence of the G-risk allele and increased susceptibility to prostate cancer; the risk allele may increase the risk of prostate cancer in its carriers by a factor of 0.449 compared to normal allele carriers (CI 95%: (0.227-0.887) OR: 0.449, p=0.02).. We examined the association of genotypes with the disease level. In this study, we assessed *rs1048943* polymorphism in men with prostate cancer. The results showed a significant correlation in Iraqi men between this polymorphism and prostate cancer. Our results agreed with other studies performed Aktas et al., (2004) and Li Li et al., (2012) .
